# Assessment of Brazilian hospital and healthcare service infrastructure for cleft lip and palate patients

**DOI:** 10.1590/1807-3107bor-2024.vol38.0103

**Published:** 2024-11-08

**Authors:** Raquel Souto Silva, Rosa Núbia Vieira de Moura, Daniele Lopes Leal, Soraia Macari, Marcos Azeredo Furquim Werneck, Rafaela da Silveira Pinto

**Affiliations:** (a) Universidade Federal de Minas Gerais – UFMG, School of Dentistry, Department of Social and Preventive Dentistry, Belo Horizonte, MG, Brazil.; (b) Universidade Federal de Minas Gerais – UFMG, School of Dentistry, Department of Restorative Dentistry, Belo Horizonte, MG, Brazil.

**Keywords:** Oral Health, Cleft Lip, Cleft Palate, Dentofacial Deformities, Dental Health Services

## Abstract

Cleft lip and palate (CLP) represent the most frequently reported congenital anomaly affecting the craniofacial region. The aim of this study was to assess the output (in number of procedures) of the Brazilian hospitals accredited for the treatment of CLP patients, examine the referral flow of patients requiring this type of care, and ascertain the adequacy of the corresponding infrastructure of these healthcare facilities. Methodologically, the study used an observational, cross-sectional, and ecological design. Output data, categorized by state and macro-region, and patient referral flow records were accessible through the Outpatient Information System (SIA, in its Portuguese acronym) and the Hospital Information System (SIH, in Portuguese), respectively. Infrastructure assessment relied on data sourced from the National Register of Health Establishments (CNES, in Portuguese). Analysis encompassed data from 28 accredited hospitals. Concerning output metrics, the state of São Paulo ranked first in the number of procedures conducted. The establishments exhibiting the lowest output performance comprised six hospitals located in the Southeast region and two in the Center-West region. Examination of patient referral flow corroborated the concentration of procedures predominantly conducted in the Southeast, notably within São Paulo state. Infrastructure evaluation encompassed the following categories: physical facilities, diagnostic and therapeutic support services, equipment, and comprehensive multidisciplinary care services. The data showed that roughly 61% of the hospitals surveyed possessed less than half of the recommended items. The primary deficiency identified pertained to inadequacies in equipment availability. Conversely, the best outcomes were associated with diagnostic and therapeutic support services. It was concluded that enhancing hospital infrastructure is imperative for the amelioration of care provision to patients with CLP across all Brazilian states.

## Introduction

Cleft lip and palate (CLP) conditions encompass deformities affecting the lip, dental arch, and palate. They rank among the most frequently reported congenital anomalies impacting the craniofacial region.^1,2^ Owing to potential implications on aesthetics, speech, hearing, swallowing, and chewing, the absence of treatment or inadequacy thereof in addressing craniofacial anomalies can precipitate significant functional and social impairments for individuals afflicted with CLP, as well as their families and broader society. Such repercussions may include morbidity, emotional disturbances, stigmatization, and social marginalization.^1,3–7^

Brazil is internationally recognized for its Unified Health System (SUS), which emerged from the Brazilian sanitary reform of 1988 and represents a collaborative effort involving policymakers, academic institutions, workers, and social movements.^8^ Enshrined within the legislation governing this system is the principle of universal and equitable access to diverse healthcare services for all Brazilian citizens. Aligned with SUS tenets of regionalization and decentralization, funding was allocated in 1993 to support the correction of cleft lip and palate within the SUS, in accordance with the Table of Procedures, Medications, Orthotics, Prostheses, and Special Materials Management System (SUS-SIGTAP).^9^ Additionally, a pivotal initiative aimed at enhancing resolution in the realm of craniofacial anomalies was the establishment of the Reference Network for the Treatment of Craniofacial Deformities (RRTDCF).^10,11^

Section VIII of Consolidation Ordinance No. 01, issued on February 22, 2022 by the Health Care Secretariat of the Ministry of Health, delineates regulations governing the registration process for hospitals conducting integrated assessment procedures with a multisectoral approach for the aesthetic and functional rehabilitation of patients with labiopalatal malformations within the SUS.^12^ Presently, there are 33 accredited centers distributed across all five regions.^13^

In this context, the aim of this study was to conduct an assessment of the output metrics of 28 accredited hospitals spanning the years 2008 to 2017 regarding the treatment of patients with CLP across Brazilian states and macro-regions, alongside an examination of patient displacement patterns when seeking specialized care. Furthermore, a descriptive analysis of the infrastructure pertaining to establishments accredited as treatment centers for lip and palate malformation by the Ministry of Health was conducted, contrasting the observed infrastructure against that recommended by the pertinent Ordinance. The goal of this endeavor was to furnish enhanced insights for the effective management and strategic planning of tertiary-level health services (hospitals) aimed at catering to the distinctive requirements of patients with orofacial clefts within the SUS framework.

## Methodology

This investigation adopted an observational, cross-sectional, and ecological design. Data spanning from January 1, 2008, to December 31, 2017, were sourced from accredited establishments recognized by the Ministry of Health, accessible via the Outpatient Information System (SIA, in its Portuguese acronym) and the Hospital Information System (SIH, in Portuguese). Data retrieval was conducted using the TabWin program provided by the Department of Informatics of the SUS (DATASUS), incorporating advanced search functionalities such as the "quantity presented" filter and the "International Classification of Diseases and Related Health Problems" (ICD-10) criteria, specifically targeting conditions associated with cleft lip and palate (codes Q35, Q36, and Q37, along with codes hierarchically related to them).

The number of procedures conducted by the hospitals was derived from data accessible through the SIA and SIH systems. Only those procedures indicating the presence of the ICD-10 codes specifically associated with cleft lip and palate as the primary diagnosis were included in this analysis, consistent with the directives outlined in Ordinance No. 1,324, dated November 27, 2014.^14^

The assessment of hospital infrastructure was conducted utilizing data extracted from the SUS information system known as the National Register of Health Establishments (CNES, in Portuguese). Various aspects pertaining to hospital infrastructure were surveyed:^12^ physical facilities, diagnostic support services, equipment availability, medical specialty services, dental services, and hospital staff. Data collection, tabulation, and analysis procedures were segregated, with one researcher conducting these activities within the SIA and SIH systems, while another researcher conducted analogous tasks within the CNES framework. MS Excel 365 software was employed for database organization, consolidation of results, and computation of percentages.

Patient referral flow records were retrieved from the SIH and SIA systems utilizing the TabWin program. The tabulation encompassed data concerning the place of residence (state) and the location of care for patients diagnosed with specified ICD-10 codes undergoing procedures related to cleft lip and palate. Subsequently, the same software was used to outline a patient referral flow map. To enhance the visual clarity of the flow map, given the tendency for line overlaps during the tabulation of actual case numbers, simplification of the tables was undertaken so that locations having at least one case were highlighted on the flow map.

The study adhered to the guidelines and regulations outlined in Resolution No. 510/2016 of the National Health Council, which governs the ethical and legal dimensions of scientific research conducted in Brazil.^15^ Given that the study exclusively relied on secondary databases, formal submission to a Research Ethics Committee was deemed unnecessary.

## Results

The study assessed output, infrastructure, and adequacy records obtained from 28 hospitals accredited for the treatment of CLP patients between 2008 and 2017, in accordance with the guidelines established by the Ministry of Health.^12^ A list of the analyzed hospitals is given in Table 1.

**Table 1:** List of healthcare establishments accredited by the Ministry of Health.

CNES		Establishment	City	State
North				
	2600536	Araguaina Hospital (*Hospital de Araguaína*)	Araguaína	TO
Northeast				
	2802104	Santo Antônio Hospital/Irma Dulce Social Works (*Hospital Santo Antônio/Obras Sociais Irmã Dulce*)	Salvador	BA
	2563681	Albert Sabin Hospital (*Hospital Albert Sabin*)	Fortaleza	CE
	0000434	Institute of Integral Medicine Professor Fernando Figueira - Maternal and Child Institute (*Instituto de Medicina Integral Professor Fernando Figueira - Instituto Materno lnfantil*)	Recife	PE
	2726998	Alcenor Almeida Piauiense Society for Fighting Cancer - São Marcos Hospital (*Sociedade Piauiense de Combate ao Câncer* Alcenor Almeida - *Hospital São Marcos*)	Teresina	PI
Southeast				
	2171988	Alzira Velano University Hospital / Alfenas Teaching and Technology Foundation (*Hospital Universitário Alzira Velano/Fundação de Ensino e Tecnologia de Alfenas*)	Alfenas	MG
	2695324	Baleia Hospital/ Benjamin Guimarães Foundation (*Hospital da Baleia/Fundação Benjamin Guimarães*)	Belo Horizonte	MG
	2269724	Our Lady of Loreto Municipal Hospital (*Hospital Municipal Nossa Senhora do Loreto*)	Rio de Janeiro	RJ
	2790564	Hospital for Rehabilitation of Craniofacial Anomalies (*Hospital de Reabilitação de Anomalias Crâniofaciais*)	Bauru	SP
	2084252	Sobrapar Campinas/Brazilian Society for Research and Assistance for Craniofacial Rehabilitation *(Sobrapar Campinas/Sociedade Brasileira de Pesquisa e Assistência para Reabilitação Crâniofacial*)	Campinas	SP
	2078015	Clinical Hospital / University of São Paulo School of Medicine Foundation (*Hospital das Clínicas / Fundação Faculdade de Medicina da Universidade de São Paulo*)	São Paulo	SP
	2786370	FUNCRAF- Foundation for the Study and Treatment of Craniofacial Deformities (*FUNCRAF - Fundação para o Estudo e Tratamento das Deformidades Craniofaciais*) *	São Bernardo do Campo	SP
	2077485	São Paulo Hospital/Paulista School of Medicine (*Hospital São Paulo/Escola Paulista de Medicina*)	São Paulo	SP
	2772310	Brotherhood of the Holy House of Mercy (*Irmandade da Santa Casa de Misericórdia*)	Piracicaba	SP
	2076039	FUNCRAF - Foundation for the Study and Treatment of Craniofacial Deformities (*Fundação para o Estudo e Tratamento das Deformidades Crânio-faciais* - FUNCRAF)*	Itapetininga	SP
	2082527	Holy House of Mercy (*Santa Casa de Misericórdia*)	Araraquara	SP
	2077396	Base Hospital of São José do Rio Preto (*Hospital de Base de São José do Rio Preto*)	São José do Rio Preto	SP
South				
	0015369	Workers’ Hospital/Support Foundation of the Federal University of Paraná (*Hospital do Trabalhador/Fundação de Apoio da Universidade Federal do Paraná*)	Curitiba	PR
	2237571	Our Lady of Conception Hospital (*Hospital Nossa Senhora da Conceição*)	Porto Alegre	RS
	2252287	Charity and Benevolence Society - Bruno Born Hospital (*Sociedade Beneficência e Caridade - Hospital Bruno Born*)	Lajeado	RS
	3508528	University Hospital of the Lutheran University of Brazil (*Hospital Universitário da Universidade Luterana do Brasil*)	Canoas	RS
	2223570	Workers’ Circle Caxiense Hospital (*Hospital do Círculo Operário Caxiense*)	Caxias do Sul	RS
	2691868	Joana de Gusmão Children's Hospital (*Hospital Infantil Joana de Gusmão*)	Florianópolis	SC
	2436450	Hans Dieter Schmidt Regional Hospital (*Hospital Regional Hans Dieter Schmidt*)	Joinville	SC
Center-West				
	2673916	Association of Social Pioneers (*Associação das Pioneiras Sociais*)	Brasília	DF
	0021709	FUNCRAF - Foundation for the Study and Treatment of Craniofacial Deformities (*Fundação para o Estudo e Tratamento das Deformidades Crânio-faciais -* FUNCRAF)*	Campo Grande	MS
	2659107	University General Hospital (*Hospital Geral Universitário*)	Cuiabá	MT
	2655411	Julio Muller University Hospital (*Hospital Universitário Júlio Muller*)	Cuiabá	MT

* Twenty-eight centers accredited by 2017, three of which only provide outpatient services (linked to FUNCRAF). The surgical procedures are conducted entirely at the Hospital for Rehabilitation of Craniofacial Anomalies in Bauru (one in São Bernardo do Campo, SP, one in Campo Grande, MS, and one in Itapetininga, SP).

Source: CGAE/DAET/SAS/MS, on April 7, 2017.

The procedures included in the output assessment of accredited establishments are detailed in Table 2, while the outcomes of these procedures during the evaluation period are depicted in Table 3. Notably, the Hospital for Rehabilitation of Craniofacial Anomalies of Bauru, SP (CNES 2790564), exhibited the highest output values across all procedures conducted, both in the SIH (36.7%) and the SIA (46.3%). These findings underscore a notable preeminence in rehabilitation procedure output within the state of São Paulo (Table 3).

**Table 2:** List of surgical and outpatient procedures performed for the treatment of CLP.

Surgical and outpatient procedures performed only in accredited centers		
	03.07.04.010-0	Installation of a prosthesis in patients with cranial and maxillofacial anomalies
	07.03.04.011-9	Fixed orthodontic/orthopedic appliance installation
	04.04.03.001-7	Columellar elongation in patients with cranial and maxillofacial anomalies
	04.04.03.003-3	Maxillary osteotomy in patients with cranial and maxillofacial anomalies
	04.04.03.004-1	Maxillofacial anomalies
	04.04.03.010-6	Primary palatoplasty in patients with cranial and maxillofacial anomalies
	04.04.03.012-2	Secondary labioplasty in patients with cranial and maxillofacial anomalies
	04.04.03.013-0	Rhinoseptoplasty in patients with cranial and maxillofacial anomalies
	04.04.03.015-7	Total lip reconstruction in patients with cranial and maxillofacial anomalies
	04.04.03.016-5	Rhinoplasty in patients with cranial and maxillofacial anomalies
	04.04.03.017-3	Septoplasty in patients with craniomaxillofacial anomalies
	04.04.03.019-0	Tympanoplasty in patients with cranial and maxillofacial anomalies (uni/bilateral)
	04.04.03.022-0	Extra-oral maxillofacial osseointegrated implant
	04.14.02.042-1	Osseointegrated dental implant
Surgical and outpatient procedures that do not require accreditation to be performed		
	03.07.04.006-2	Periodic maintenance of oral and maxillofacial prosthesis
	04.04.03.005-0	Mandible osteotomy in patients with craniomaxillofacial anomalies
	04.04.03.006-8	Chin osteoplasty with or without alloplastic implant
	04.04.03.007-6	Two-stage unilateral labioplasty
	04.04.03.008-4	Alveoloplasty with bone graft in patient with craniofacial anomalies
	04.04.03.024-6	Surgical treatment of oronasal fistula in patients with anomalies
	04.04.03.025-4	Surgical treatment of oronasal fistulas in patients with anomalies
	04.04.03.026-2	Secondary palatoplasty in patients with cranial and maxillofacial anomalies
	04.04.03.027-0	Surgical treatment of velopharyngeal insufficiency in patients with craniomaxillofacial anomalies
	04.04.03.028-9	Restorative surgical treatment of rare facial cleft in patients with cranial and maxillofacial anomalies
	04.04.03.029-7	Complex craniofacial osteotomy in patients with craniofacial, oral and maxillofacial anomalies
	04.04.03.030-0	Craniofacial remodeling in patients with craniofacial, oral and maxillofacial anomalies
	04.04.03.031-9	Surgical treatment of macrostomia/microstomia due to anomalies
	04.04.03.032-7	Fronto-orbital osteoplasty
	04.04.02.027-5	Resection of malignant and benign lesions in cranial and maxillofacial region
	04.14.01.037-0	Surgical treatment of impacted tooth in patients with cranial and maxillofacial anomalies
	04.14.02.035-9	Surgical treatment of oral bleeding
	04.15.02.004-2	Sequential procedures in cranial and maxillofacial anomalies
	07.01.08.004-3	Fixed prosthesis in patients with cranial and maxillofacial anomalies
	07.01.08.009-4	Removable prosthesis in patients with cranial and maxillofacial anomalies
	07.01.08.011-6	Mandibular prosthesis
	07.01.08.012-4	Prosthesis for extensive maxillary losses
	07.01.08.013-2	Implant-supported ear prosthesis
	07.01.08.014-0	Extensive facial prosthesis (2/3 of the face)
	07.01.08.015-9	Implant-supported prosthesis for large maxillary loss
	07.01.08.016-7	Implant-supported lip prosthesis
	07.01.08.017-5	Implant-supported nasal prosthesis
	07.01.08.018-3	Implant-supported ocular-palpebral prosthesis
	07.01.08.019-1	Implant-supported palatopharyngeal obturator prosthesis
	07.01.08.004-3	Fixed prosthesis in patients with cranial and maxillofacial anomalies
	07.01.08.005-1	Prosthesis for extensive maxillary loss
	07.01.08.006-0	Lip prosthesis
	07.01.08.007-8	Nasal prosthesis
	07.01.08.008-6	Ocular-palpebral prosthesis
	07.01.08.009-4	Removable prosthesis in patients with cranial and maxillofacial anomalies
	07.01.08.002-7	Ear prosthesis

**Table 3:** Hospital and outpatient output of accredited hospitals.

		Hospital Information System - SIH									Outpatient Information System - SIA														
CNES		Year of attendance										Total	%	Year of attendance										Total	%
		2008	2009	2010	2011	2012	2013	2014	2015	2016	2017			2008	2009	2010	2011	2012	2013	2014	2015	2016	2017		
**North**		**25**	**21**	**26**	**33**	**34**	**18**	**25**	**2**	**3**	**23**	**210**	**0.67**	**0**	**0**	**0**	**2**	**0**	**0**	**0**	**6**	**0**	**14**	**22**	**0.03**
	2600536	25	21	26	33	34	18	25	2	3	23	210	0.67	0	0	0	2	0	0	0	6	0	14	22	0.03
**Northeast**		**599**	**720**	**711**	**723**	**842**	**634**	**740**	**691**	**759**	**597**	**7,016**	**22.3**	**36**	**49**	**74**	**570**	**1,075**	**1,264**	**1,231**	**1,044**	**992**	**1,067**	**7,402**	**11.27**
	2802104	126	126	170	170	117	129	141	116	100	100	1,295	4.12	2	22	44	6	81	253	152	152	96	215	1,023	1.56
	2563681	27	154	184	128	285	142	209	240	269	194	1,832	5.82	0	0	0	33	256	270	319	127	267	234	1,506	2.29
	0000434	304	327	241	307	291	211	267	241	259	219	2,667	8.48	26	14	26	326	484	476	564	560	467	442	3,385	5.15
	2726998	142	113	116	118	149	152	123	94	131	84	1,222	3.88	8	13	4	205	254	265	196	205	162	176	1,488	2.26
**Southeast**		**2,028**	**1,821**	**1,598**	**2,076**	**1,706**	**1,828**	**1,871**	**1,977**	**1,68**	**1,308**	**17,893**	**56.86**	**679**	**711**	**592**	**7,461**	**7,133**	**7,345**	**6,337**	**6,467**	**5,318**	**4,743**	**46,786**	**71.21**
	2171988	28	38	42	82	78	96	90	91	77	68	690	2.19	4	9	4	10	39	9	15	3	163	53	309	0.47
	2695324	137	137	124	125	100	114	130	154	117	106	1,244	3.95	3	5	4	8	20	11	25	11	1	9	97	0.15
	2269724	124	97	133	153	137	199	171	129	147	116	1,406	4.47	0	0	0	395	659	352	411	678	539	284	3,318	5.05
	2790564	1,428	1,266	1,084	1,421	1,092	1,133	1,168	1,276	1,034	648	11,55	36.7	561	583	472	4,878	4,631	5,093	4,087	4,411	3,023	2,671	30,41	46.29
	2084252	236	237	170	194	210	180	189	237	215	271	2,139	6.80	0	0	0	405	300	433	371	386	500	507	2,902	4.42
	2078015	67	41	40	85	74	88	96	71	70	79	711	2.26	0	0	0	1	20	10	28	17	17	16	109	0.17
	2786370*	0	0	0	0	0	0	0	0	0	0	0	0	111	114	112	1,616	1,361	1,343	1,287	851	953	1,098	8,846	13.46
	2077485	6	3	3	13	13	15	19	13	12	10	107	0.34	0	0	0	0	0	0	0	0	0	0	0	0.00
	2772310	2	2	2	3	1	0	1	1	0	1	13	0.04	0	0	0	0	0	0	0	0	0	0	0	0.00
	2076039*	0	0	0	0	0	0	0	0	0	0	0	0.00	0	0	0	123	100	94	113	109	122	105	766	1.17
	2082527	0	0	0	0	1	0	0	0	0	2	3	0.01	0	0	0	25	3	0	0	0	0	0	28	0.04
	2077396	0	0	0	0	0	3	7	5	8	7	30	0.10	0	0	0	0	0	0	0	1	0	0	1	0.00
**South**		**606**	**624**	**510**	**576**	**645**	**694**	**622**	**545**	**392**	**405**	**5,619**	**17.86**	**75**	**121**	**123**	**1,187**	**1,392**	**1,369**	**1255**	**1,363**	**1,418**	**1,342**	**9,645**	**14.68**
	0015369	354	389	299	268	252	222	234	190	141	146	2,495	7.93	16	68	81	499	367	379	496	527	605	574	3,612	5.50
	2237571	20	46	42	28	52	81	73	85	76	85	588	1.87	0	0	0	0	4	0	1	0	0	0	5	0.01
	2252287	121	97	85	131	151	197	136	149	122	93	1,282	4.07	23	5	6	582	692	702	531	630	604	561	4,336	6.60
	3508528	0	0	2	1	1	4	1	11	6	6	32	0.10	0	0	0	0	0	0	0	0	0	0	0	0
	2223570	0	0	0	0	23	27	44	42	37	42	215	0.68	0	0	0	0	95	67	29	11	8	6	216	0.33
	2691868	15	7	6	14	9	12	13	14	10	33	133	0.42	0	0	0	0	0	0	0	0	0	0	0	0.00
	2436450	96	85	76	134	157	151	121	54	0	0	874	2.78	36	48	36	106	234	221	198	195	201	201	1,476	2.25
**Center-West**		**15**	**58**	**50**	**56**	**68**	**72**	**104**	**86**	**129**	**92**	**730**	**2.32**	**0**	**70**	**50**	**453**	**199**	**199**	**230**	**221**	**212**	**210**	**1,844**	**2.81**
	2673916	0	0	0	0	0	0	0	0	0	0	0	0.00	0	0	0	0	0	0	0	0	0	0	0	0
	0021709*	0	0	0	0	0	0	0	0	0	0	0	0.00	0	70	50	453	199	194	207	193	162	187	1,715	2.61
	2659107	12	56	49	48	49	44	61	69	65	64	517	1.64	0	0	0	0	0	5	23	26	49	17	120	0.18
	2655411	3	2	1	8	19	28	43	17	64	28	213	0.68	0	0	0	0	0	0	0	2	1	6	9	0.01
**BRAZIL**		**3,273**	**3,244**	**2,895**	**3,464**	**3,295**	**3,246**	**3,362**	**3,301**	**2,963**	**2,425**	**31,468**	**100.00**	**790**	**951**	**839**	**9,673**	**9,799**	**10,177**	**9,053**	**9,101**	**7,94**	**7,376**	**65,699**	**100**

* Twenty-eight centers accredited by 2017, three of which only provide outpatient services (linked to FUNCRAF). The surgical procedures are conducted entirely at the Hospital for Rehabilitation of Craniofacial Anomalies in Bauru (one in São Bernardo do Campo, SP, one in Campo Grande, MS, and one in Itapetininga, SP).

Source: CGAE/DAET/SAS/MS, on April 7, 2017.

The findings pertaining to the infrastructure guidelines recommended by the Ministry of Health are shown in Table 4, categorized into the following domains: physical facilities, diagnostic and therapeutic support services, equipment, and comprehensive care multidisciplinary services. As stipulated in the pertinent Ordinance, the professional prerequisites encompassed possession of a specialist title in the relevant area, registration with the professional board, and evidence of training in the requisite professions. However, the registration forms of the establishments lacked pertinent information regarding the qualification of professionals. Consequently, although stipulated in the Ordinance, this aspect was omitted from the study's analysis.

**Table 4:** Infrastructure items recommended by Section VIII of Consolidation Ordinance No. 01, dated February 22, 2022.

Variables	
Equipment (n = 15)	
	Audiometer;
	Impedance meter;
	Xray machine for cephalometric radiography and orthopantomography;
	Xray unit for periapical and occlusal radiography;
	Nasopharyngoscope;
	Videofluoroscopy;
	Device with micromotor with speed control;
	Ultrasonic device for cleaning instruments;
	Autoclave;
	Dental chair with foot control;
	Supplies for implantintegrated bone systems;
	Vacuum suction unit;
	Electric saw for craniofacial surgery;
	Frontal illuminator (focus);
	Supplies for maxillomandibular fixation.
Physical facilities (n = 18)	
	Inpatient unit for children and adults;
	Surgical center equipped with oxygen, respirator, nitrous acid, cardiac monitor, electric scalpel, defibrillator, and anesthesia cart;
	Recovery room inside the surgical building equipped with cardiac monitor and defibrillator, in addition to other supplies and medications necessary for cardiorespiratory emergencies;
	Room for minor surgery (dentistry);
	Dental offices equipped with Xray device and appropriate equipment to perform oral and maxillofacial surgery and implant placement (sterilizers and contraangle with speed control);
	Soundproof booth for speech therapy;
	Room for videofluoroscopy.
Diagnostic and therapeutic support service (n = 6)	
	Laboratory of clinical pathology;
	Laboratory of prosthesis;
	Laboratory of orthodontics;
	Nosocomial infection control commission;
	Patient records service;
	Documentation service capable of documenting treatment sequence using slides, photographs, and dental Xrays.
Multidisciplinary Comprehensive care services (n = 17)	
	Anesthesia;
	Aesthetic plastic surgery;
	Otorhinolaryngology;
	Medical clinic;
	Pediatrics;
	Speech therapy;
	Psychology;
	Physiotherapy;
	Nursing;
	Social service;
	Nutrition;
	General dentistry;
	Pediatric dentistry;
	Orthodontics;
	Prosthesis and Implant dentistry;
	Oral and maxillofacial surgery;
	Family assistance.
Professionals (n = 4)	
	Medical and dental professionals responsible for specific services having a specialist degree in the related area.
	Prosthodontists duly registered with the Federal Board of Dentistry and having experience in extraoral prostheses.
	Dental assistants and/or hygienists duly registered with the Federal Board of Dentistry or equivalent recognized capacity.
	Speech therapy professionals having evidence of training totaling at least 320 hours over a period of 2 months.

The outcome of the infrastructure assessment conducted on accredited hospitals is summarized in Table 5. According to the infrastructure data obtained through the CNES, it was revealed that 17 out of the 28 hospitals (approximately 61%) possessed less than half of the items recommended by the Ministry of Health. Notably, the primary deficiency observed pertained to equipment, with records from three hospitals indicating the absence of any equipment listed in the Ordinance. Additionally, another component exhibiting suboptimal compliance was physical facilities, with 13 hospitals (46%) possessing less than half of the recommended items in this category. Conversely, Diagnostic and Therapeutic Support Services yielded more favorable outcomes, with 68% of hospitals possessing more than four out of the six stipulated items. Remarkably, presence of a Hospital Infection Control Commission (CCIH) was documented in 100% of the records across all hospitals (Table 5). The patient referral flow map outlined based on the surveyed data is depicted in Figure.

**Figure 1:**
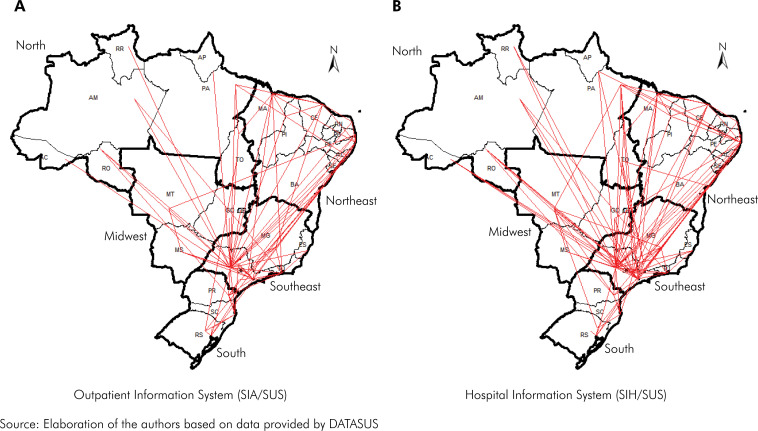
Displacement flow of patients with ICD-10 related to cleft lip and palate to perform procedures that require or do not the authorization of establishments to perform them, according to the Outpatient and Hospital Information Systems of the SUS, 2008-2017.

**Table 5:** Infrastructure assessment according to compliance with Section VIII of Consolidation Ordinance No. 01, dated February 22, 2022.

CNES	Physical facilities (n = 18)	Diagnostic support services (n = 6)	Equipment (n = 15)	Services (n = 17)	Professionals (n= 4)	Total (n = 61)
	%	%	%	%	%	%
2802104	52.63	66.67	20.00	82.35	0.00	50.82
2563681	52.63	66.67	6.67	76.47	0.00	45.90
2673916	42.11	66.67	0.00	52.94	0.00	34.42
2171988	47.37	66.67	20.00	88.24	0.00	50.82
2695324	42.11	66.67	6.67	88.24	0.00	45.90
0021709	5.26	33.33	13.33	64.71	0.00	26.23
2659107	47.37	50.00	13.13	88.24	0.00	47.54
2655411	36.84	66.67	6.67	76.47	0.00	40.98
0000434	57.89	66.67	13.33	88.24	0.00	52.46
2726998	57.89	66.67	13.33	94.12	0.00	54.10
0015369	57.89	66.67	20.00	94.12	0.00	55.74
2269724	47.37	50.00	20.00	76.47	0.00	45.90
2237571	57.89	66.67	20.00	94.12	0.00	55.74
2252287	57.89	66.67	20.00	94.12	0.00	55.74
3508528	52.63	66.67	6.67	88.24	0.00	49.18
2223570	47.37	66.67	0.00	88.24	0.00	45.90
2691868	52.63	66.67	13.33	76.47	0.00	47.54
2436450	47.37	66.67	6.67	94.12	0.00	49.18
2790564	47.37	66.67	20.00	94.12	0.00	52.46
2084252	52.63	50.00	20.00	64.71	0.00	44.26
2078015	57.89	66.67	20.00	88.24	0.00	54.10
2786370	10.53	33.33	20.00	64.71	0.00	29.51
2077485	57.89	50.00	26.67	70.59	0.00	49.18
2772310	57.89	33.33	20.00	64.71	0.00	44.26
2076039	5.26	16.67	13.33	47.06	0.00	19.67
2772310	47.37	50.00	0.00	82.35	0.00	42.62
2077396	57.89	50.00	20.00	88.24	0.00	52.46
2600536	57.89	66.67	13.33	82.35	0.00	50.82

* Twenty-eight centers accredited by 2017, three of which only provide outpatient services (linked to FUNCRAF). The surgical procedures are conducted entirely at the Hospital for Rehabilitation of Craniofacial Anomalies in Bauru (one in São Bernardo do Campo, SP, one in Campo Grande, MS, and one in Itapetininga, SP).

Source: CGAE/DAET/SAS/MS, on April 7, 2017.

## Discussion

This study revealed that, with regard to the infrastructure and adequacy of the 28 hospitals accredited by the SUS for the rehabilitation of patients with labiopalatal malformations until the year 2017, the primary deficiencies identified pertained to equipment and physical facilities. Conversely, the aspects exhibiting better coverage were Diagnostic and Therapeutic Support Services, along with the presence of a CCIH. Moreover, the hospitals with the highest volume of CLP procedures were situated in the Southeast region of Brazil.

Guidelines and parameters defining care for patients with CLP are referenced in the related literature.^16,17^ In Brazil, implementation of the RRTDCF within the SUS is governed by regulations stipulating semiannual evaluations of registered hospitals’ performance. The findings from these evaluations are subsequently forwarded to State Health Secretariats and to the Hospital for Rehabilitation of Craniofacial Anomalies of Bauru, for review and advisement.^12^ The results that were found to be below the established benchmarks underscore the necessity of adhering to this health surveillance proposal.^18^ Accordingly, the involved establishments were shown to integrate a service network that remains insufficient and inconsistently distributed across the Brazilian population. Despite the considerable number of facilities, regional disparities persist in the provision of care for CLP patients in Brazil.^10^

The healthcare establishments with the poorest performance comprised six hospitals in the Southeast region and two in the Center-West region. Given the significant concentration of facilities in the Southeast region, the underperformance of some of these establishments is not unexpected. However, the limited output observed in the Center-West region may be attributed to inadequate infrastructure, characterized by hospitals possessing few mandatory equipment items, due to the unequal allocation of resources, an issue documented in previous studies.^19,20^ The inadequacy of establishments represents a significant public health concern.^18^ When comparing output with the infrastructure of establishments that demonstrate a predominance in national productivity, the Hospital for Rehabilitation of Craniofacial Anomalies of Bauru, SP, exhibited deficiencies in physical facilities and equipment listed in the CNES registration form. However, concerning diagnostic support services and professional staff comprising specialists recommended by the pertinent Ordinance, it demonstrated performance exceeding 50%, ranking seventh when considering all aspects of infrastructure adequacy. Organizing the infrastructure of accredited hospitals is crucial to enable them to address patient demands effectively, thereby preventing extensive travel or treatment abandonment.^10^

Although there is a notable concentration of Rehabilitation Centers in the South and Southeast regions,^21,22^ this observation aligns with studies indicating a higher prevalence of live births with CLP in the South region of the country.^10,11^ Establishments situated in the South and Southeast regions exhibited superior performance in this study concerning infrastructure and contributed significantly to overall output, collaborating extensively with centers in other regions. Specifically, over two-thirds of outpatient output (71.21%) and more than half of hospital output (56.86%) originated from the Southeast region. The limited availability of services in the North (only one institution), Northeast (four institutions), and Center-West (four institutions) regions, coupled with centers exhibiting inadequate output levels, may necessitate significant travel for users seeking care, often requiring them to visit centers located far from their place of residence.^21^ This trend is evident in the patient referral flow map, which illustrates the location of different Brazilian centers and a notable concentration of flow lines directed towards the Southeast, particularly the state of São Paulo. Additionally, a less pronounced flow can be seen towards the Northeast, primarily among northeastern states and certain northern states.

Emphasis should be placed on the Hospital for Rehabilitation of Craniofacial Anomalies of Bauru, SP, which serves as a pivotal institution providing services for numerous other states and holds national recognition as a center of excellence. The flow map clearly illustrates the extensive movement of patients from all over the country seeking care at this center, notwithstanding the presence of accredited hospitals in certain states. Despite the existence of 28 centers across 13 states within the country, some are limited to serving local or regional populations exclusively. Thus, it is anticipated that, through the appropriate allocation of requisite resources and the optimization of operational workflows, a more decentralized, qualified, and effective response can be achieved to meet the healthcare needs of populations residing in their respective states.

The necessity for new healthcare facilities and the adequacy of existing services warrant thorough examination. In the state of São Paulo, for instance, there are nine accredited hospitals, the majority of which exhibit limited output and encounter challenges in adapting their infrastructure to comply with regulatory standards applicable to such establishments. It is imperative for governmental authorities to conduct technical assessments to ascertain the requirement for new services and to evaluate the quality of those already in operation. In this regard, Almeida and Chaves^18^ developed an evaluative framework for CLP care, comprising two dimensions: a) care management, encompassing the presence of a multidisciplinary team, infrastructure, supplies, protocols, and registration systems; and b) patient rehabilitation, delineating the roles and responsibilities of healthcare professionals engaged in CLP rehabilitation. This model holds promise for guiding these evaluations.

The geographic dimension plays a pivotal role in shaping the organization of public health systems.^10^ Despite the considerable number of healthcare centers catering to patients with CLP across Brazil, discernible regional disparities persist in both their geographic dispersion and output capacity. Such disparities may precipitate delays in diagnosis or result in untreated cases. Moreover, the incongruence between service provision, the necessity to traverse vast distances, and the associated high travel costs may compel many families to forgo treatment or follow-up care offered by these centers.^10,19,24^ Consequently, children lacking appropriate management may encounter difficulties in performing routine activities such as feeding, speech development, or achieving weight gain consistent with healthy child development.^23^

A limitation of the study stems from its reliance on secondary data derived from the completion of CNES registration forms, on data provided by the SIH and SIA systems, as well as on the identification of ICD-10 codes recorded in Hospital Admission Authorizations (a tool utilized for recording information within the SIH). The configuration of services has a demonstrable impact, particularly when integrated with a national registration and research strategy.^25^. Despite the increasing availability of studies utilizing such secondary data sources, the reliability of the information hinges upon the completeness of data and accurate notifications.^26,27^ Additionally, it is noteworthy that the SIH and SIA systems record the number of hospitalizations rather than the number of individual patients ^10,28^. This limitation can pose challenges in analyzing and processing the data, particularly concerning conditions such as CLP, where individuals may undergo multiple surgeries. Despite these acknowledged limitations, data extraction was conducted by a researcher specializing in information from the SIA and SIH systems to mitigate potential biases in the study.

## Conclusion

The data provided by this study can contribute to inform the redistribution of resources, ensure the adequacy of services provided by healthcare facilities in accordance with the epidemiological needs of users, and facilitate the effective regionalization of services offered to patients with CLP across the country. There is a pressing need to enhance the healthcare network for patients with CLP, ensuring equitable allocation of services and optimizing the organization of care pathways, with the ultimate goal of establishing comprehensive guidelines for this specialized line of care. Furthermore, it is imperative to upgrade the infrastructure of healthcare facilities, informed by the strengthening of health surveillance efforts, to ensure compliance with the requirements outlined in Ordinance No. 62 of April 19, 1994.
